# Supplementary material for: Examining the impact of social media usage on start-ups performance: Mediating role of brand image
Source: PLoS One. 2025 May 14;20(5):e0320133. doi: 10.1371/journal.pone.0320133 (PMC12077680; doi:10.1371/journal.pone.0320133)
Supplement: Attachment — Submitted filename: Authors respond to PLOS ONE Reviewer COMMENT.docx [file pone.0320133.s002.docx]

Reviewer #1: The manuscript showed a technically sound paper of scientific research with data that supports the conclusions. The paper aimed to explore the impact of social media usage to startup performance with a mediating role of brand image. Data collected by sending the questionnaires to startup firms in Ghana and have 450 respondents in valid. The paper show the respondents profile, conducted the factor loading, Cronbach's Alpha, CR, VIF, AVE and Fornell-Larcker for measurement variables; hypothesis testing by PLS-SEM. The discussions, conclusions and implications were drawn appropriately based on the data collected.

The statistical analysis was performed approciately and rigorously.

Data collected are permitted from National Board for Small-Scale Industries (NBSSI) and the respondents agreed to participating. However, the data was not divided into the direct responses and google form responses. The paper has not showed how to choose the start-ups to sending the questionnaire. So that, the authors should be show the data more clearly in this points.

The manuscript was showed in intelligible fashion and written in standard English. However, the paper should considerably the form of citation. The citation based on the number of references made the target readers to have to check "what is it", eg. line 35 "From a start-up perspective, [15] noted...." the readers don't know what is [15]; or line 40 "According to [17],..." what is [17],... there have a lot of above-mentioned citations.

The paper is too long, the authors should be rewrite for shorter.

Reviewer #2: I consider the article very interesting and solid. A few adjustments to the language and the text should be made. For example:

59-60 It is not clear what you wanted to say. Seems distinct from the aim stated in the title and the rest of the paper. And probably would sound better with ”the relationship between ...”

*Thanks for the comment.* *We agree with the reviewer and have explained how direct responses and google form were filled by the responses, as shown in the manuscript (methodology section)*

*“Out of the 520 questionnaires administered, 450 of the questionnaires received were valid for data processing and analysis. The questionnaires were administered via Goggle Forms (i.e. through emails and social media platforms such as Facebook Messenger and WhatsApp) and offline methods after formal approval from the selected young entrepreneurs. Additionally, young entrepreneurs were chosen based on those who utilizes social media networks for their business activities and as a marketing communication tool”.*

*Again, issues related to citations in the manuscript has been resolved and rewritten to sound well.*

*For citation number 15 for instance “As summarized by [15-17] for instance, young entrepreneurs have started utilizing social media to facilitate effective communication with consumers and business visibility. As a result, social media adoption can lead to double profits, which boosts marketing effectiveness [4].*

*For citation number 35 “In investigating social media usage and young entrepreneur investment, Chen and Liu [35] revealed how social media has become an important marketing tool for entrepreneurship activities.*

*For citation number 40 “Moreover, other researchers [31, 35, 40] have employed the research-based view theory to investigate the impact of social media on start-up performance and sustainability.*

*For citation number 59 and 60 have been rewritten “Okrah et al. [57] focus on factors of startup success and growth by arguing that social media technologies are taking over the Ghanaian business landscape to drive sustenance, especially among small and medium enterprises. Several works have established that social media enables startups in Ghana to connect the entire ecosystem [59, 60], sharing knowledge [61] and networking [62].*

*Besides, the overall manuscript has been rewritten to make it shorter as requested.*

*Furthermore, issues related with 382 - Indicator 1 of construct IC and Construct SP indicators, the author have tackled this issue, dwelling on innovation capabilities and brand image, presented in the manuscript (literature review), developing hypothesis for it and removed all the notes on mediating role of brand image. Besides, the authors have provided discussion for the relationship between innovation capabilities and brand image in the manuscripts “Lastly, the study also considered establishing the relationship between innovation capabilities and brand image. The findings of the proposed hypothesis were supported and therefore confirm the works of [94]. According to a study by Amoah et al. [128], innovation has a significant impact on brand image hence affirming a positive relationship between innovation capabilities and the perception of brand image. Innovation capabilities empower companies to create distinct processes and brands, ultimately aiming to achieve a competitive advantage. For instance, Halliday and Trott [127] concluded that there is a positive relationship between innovation capabilities and brand image. Thus, companies can enhance their brand image by focusing on innovation mechanisms or managing design innovation effectively. [86] reinforced that firms with innovation capabilities create distinctive processes and products that enhance their brand image. For instance, [86], and Amoah et al., [128] concluded that high levels of innovation are beneficial for brand-focused in building a powerful brand image. Additionally, companies that prioritize technological and design innovation management significantly improve their brand image, further reinforcing a positive link between innovation capabilities and brand image. As indicated by [95] fostering innovation do not only differentiate a company’s products and services but also strengthens its brand image in the competitive market landscape”.*
